# Oral administration of injectable vitamin K1 in brodifacoum intoxication

**DOI:** 10.37796/2211-8039.1305

**Published:** 2022-06-01

**Authors:** Yen-Jung Chu, Jing-Hua Lin, Dong-Zong Hung

**Affiliations:** a Department of Emergency Medicine, China Medical University Hospital, Taichung, Taiwan; b Division of Toxicology, China Medical University Hospital, Taichung, Taiwan; c Graduate Institute of Biomedical Sciences, China Medical University, Taichung, Taiwan

**Keywords:** Brodifacoum, Rodenticide, Oral administration, Vitamin K1

## Abstract

Brodifacoum is a highly potent and long-acting anticoagulant rodenticide (LAAR). LAAR poisoning possibly leads to long term bleeding problems and needs vitamin K1 treatment for several months. Due to economic concern, tablet preparation of vitamin K1 was not available in most of the countries, including Taiwan. In literature, few reports had pointed out that injectable form of vitamin K could be used orally in patients on anticoagulant therapy with supratherapeutic state. Here, we reported a family with 3 members suffering from brodifacoum poisoning with severe coagulopathy needed to prolong hospitalization for intravenous vitamin K1 therapy, and successfully managed with injectable formula orally for about 5 months. Oral administration of injectable vitamin K1 might be a suitable substitute for intravenous route in long-term treatment for LAAR poisonings.

## 1. Introduction

Anticoagulant compounds are the most common used rodenticides in the world. Warfarin had been introduced for rodenticide use in the 1940s, and gradually replaced by “super-warfarins” due to the emergence of warfarin-resistant rats. Superwarfarins are highly potent vitamin K antagonists, as nominated due to being much more potent and long acting than warfarin, so called long-acting anticoagulant rodenticide (LAAR). Common examples of LAAR include brodifacoum, bromadiolone, and chlorophacinone. Long-acting anticoagulant rodenticides have an extremely high affinity for vitamin K epoxide reductase (VKOR), a key enzyme in liver for vitamin K reactivation, compared with warfarin, characterized by rebound coagulopathy and bleeding after initial treatment and the need for high-dose, long-term therapy of vitamin K [1].

Exposure to these rodenticides, mostly by oral route, can result in potentially fatal hemorrhage. Therefore, a prolonged prothrombin time as indicated with elevated international normalized ratio (INR) caused by LAAR intoxication warrants vitamin K1 treatment. The tissue half-lives of LAARs estimated at the range of 16 to 220days, and vitamin K1 administration is usually needed for extended periods of time, ranging from 28 to 730 days in the maintenance phase of treatment [1–3]. Vitamin K1 is fat-soluble and preferred being used intravenously in the newborns for bleeding tendency prevention, also in cases of supra-therapeutic state of anticoagulant and in LAAR poisonings.

Oral vitamin K to reverse the coagulopathy induced by warfarin and LLARs is being effective and promising. But, the rising cost of vitamin K tablet formulation had resulted it to be unavailable in most of the countries due to economic concern, including Taiwan [1]. Oral administration of the less expensive injectable vitamin K1 has been suggested in the newborns or in supra-therapeutic state of anticoagulant therapy [4,5]. The compounded oral solution showed increased bioavailability as compared with the tablet formulation. In addition, oral vitamin K solution has been shown to have efficacy similar to oral tablets relative to the reversal of vitamin K antagonists for patients with an elevated INR [6]. In an outbreak of patients poisoned with long-acting anticoagulant rodenticide-tainted synthetic cannabinoids (LAAR-SC) in 2018, oral administration of injectable vitamin K1 had been suggested due to shortage of oral form of vitamin K1 [7]. Here, we reported a family with 3 members suffered from brodifacoum poisoning with severe coagulopathy needed for prolong hospitalization and intravenous vitamin K1 therapy. We prescribed injectable vitamin K1 formula orally substituted intravenously for them after restoring their coagulation ability and can follow-up successfully on outpatient service.

## 2. Case report

A 45-year-old mother (65 KG-BW) brought her two sons (age 18 and 15) to our emergency department with chief complaints of epistaxis and hematuria for 2–3 days. The mother confessed that she blended 500 mL of Brodifacoum solution (0.005% w/w) into the fried rice and fruit juice, and shared in dinner about 10 days before their visit. The mother suffered from hematuria and epistaxis. The elder son (82 KG-BW), a case of poliomyelitis history and ambulatory with self-controlled wheelchair, developed mild fever, left flank pain and hematuria. The second son (74 KG-BW) suffered from epistasis, gum bleeding, abdominal pain, and tarry stool. Their initial prothrombin times/INRs all were greater than the maximal measurable value (>81 s/9.6), and prolongation of activated partial thromboplastin times (aPTT) were also noted.

**Fig. 1:**
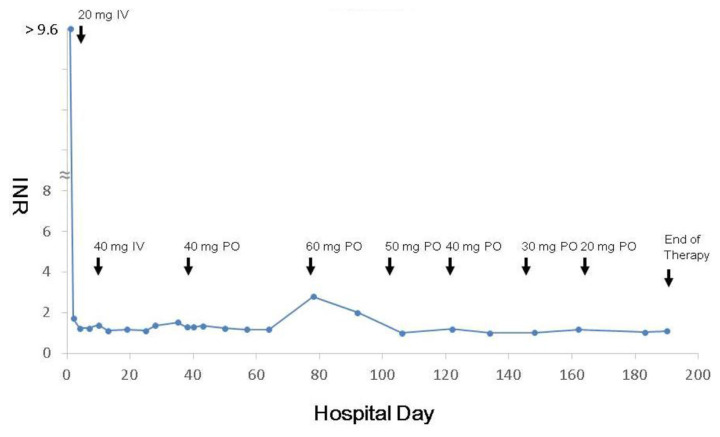
Serial INR and doses of vitamin K1 of the 15 y/o boy. He initially presented epistasis, gum bleeding, abdominal pain, and tarry stool. Poor compliance was noted between days 63 and 77. IV: intravenous route; PO: oral route.

After fresh frozen plasma transfusion and vitamin K1 intravenous injection, they were admitted for more doses of vitamin K1. No more bleeding tendency with normal or near normal INR were noted under 30 mg to 40 mg of vitamin K1 treatment in 3 days. But, rebounding of INR and prolonged hospitalization were noted if we tried to taper the dose of vitamin K1 (Fig. 1). We started to try oral administration of injectable vitamin K1 preparation 50 mg to 60 mg daily later and adjusted the dose by serial INR examinations in the outpatient department. The undiluted vitamin K1 preparation is 10 mg/mL stored in a light-proof sterilized glass bottle and refrigerator. We prepared the fresh compounds every two weeks. They reported absence of adverse effects, except for the unpleasant taste of the oily fluid. After 145 days of oral vitamin K1 therapy, they recovered with normal INR and needed no more medication again.

## 3. Discussion

These 3 patients presented typical presentations about 2 weeks after LAAR large dose exposure. Their blood tests of PT, aPTT and INR returned to normal and no more bleeding tendency shortly after fresh frozen plasma transfusion and vitamin K1 prescription. In animal studies, the serum half-life of vitamin K1 is about 6 h, and in human studies, the vitamin K dependent coagulation factors in blood are to rise at 6 to 8 h after therapy [7]. Most of the LAARs are highly lipid soluble and increased affinity to hepatic tissue and hepatic enzymes, making them to be 100 times more potent and longer acting than warfarin. The great disparate half-lives of vitamin K1 and LAARs makes it possible that recurrent symptoms or abnormalities in coagulation assays develop in 12 to 16 h after initial treatment. Thus, chronic maintenance therapy with vitamin K1 should be promptly initiated and may be required for several months [1]. So, the doses of vitamin K1 to keep normal INRs for these patients were initially titrated to 30 mg to 40 mg/day in divided doses.

Vitamin K1 can be administered through oral, intramuscular, subcutaneous, and intravenous routes; no comparing studies of the different efficacy on LAAR poisoning in literature. On the other hand, in warfarin reversal study, Intravenous administration could correct more quickly than oral route within 6 to 12 h, instead of 24 h [1]. However, potential anaphylactoid reaction with intravenous administration and hematoma formation with intramuscular injection of oily vitamin K1 in LAAR poisoning were concerned frequently. In addition, the tablet formulation of vitamin K1 is difficult to obtain or unavailable due to the increased production cost in many countries, also in Taiwan. One option to resolve these problems for patient care might be using a less expensive formulation of injectable vitamin K1 as an alternative for LAARs poisoning.

In literature, oral administration of injectable vitamin K1 has been shown to be effective and safe [4,5]. Crowther and colleagues found that administering injectable vitamin K1 orally might be more effective than subcutaneous vitamin K1 for INR reversing in patients under warfarin therapy with INRs between 4.5 and 10 [4]. In healthy volunteer trial, Van Rein et al. discovered the bioavailability of 5-mg Vitamin K1 solution was greater than that of tablet form. They also found that vitamin K1 oil solutions decreased slightly more INR than tablets in patients on anticoagulant therapy with an INR of 7.0–11.0 [5]. The stability of injectable vitamin K1 solution for oral purpose has also been studied. In literature review by Afanasjeva [6], several preparations have been mentioned; including undiluted injectable vitamin K or compounded oral solution. He found that the fluid’s preparation stability ranges from 30 days to 111 days under both room and refrigerated temperatures when protected from light. The unpleasant taste of undiluted vitamin K1 can be eliminated when mixed with orange juice.

## 4. Conclusion

While tablet formula or preparation of vitamin k1 is generally not accessible, oral administration of injectable vitamin K1 appears to be an effective and safe way for maintaining long-term therapy of LAARs poisoning in out-patient clinics to keep suitable INR. More investigations are needed to clarify the pharmacokinetics of injectable vitamin K1 from oral route and its effect on toxicology of LAARs.
